# Results on the spatial resolution of repetitive transcranial magnetic stimulation for cortical language mapping during object naming in healthy subjects

**DOI:** 10.1186/s12868-016-0305-4

**Published:** 2016-10-24

**Authors:** Nico Sollmann, Theresa Hauck, Lorena Tussis, Sebastian Ille, Stefanie Maurer, Tobias Boeckh-Behrens, Florian Ringel, Bernhard Meyer, Sandro M. Krieg

**Affiliations:** 1 Department of Neurosurgery, Klinikum rechts der Isar, Technische Universität München, Ismaninger Str. 22, 81675 Munich, Germany; 2 TUM-Neuroimaging Center, Klinikum rechts der Isar, Technische Universität München, Ismaninger Str. 22, 81675 Munich, Germany; 3 Section of Neuroradiology, Department of Radiology, Klinikum rechts der Isar, Technische Universität München, Ismaninger Str. 22, 81675 Munich, Germany

**Keywords:** Cortical stimulation, Language function, Navigated transcranial magnetic stimulation, Preoperative language mapping, Spatial resolution

## Abstract

**Background:**

The spatial resolution of repetitive navigated transcranial magnetic stimulation (rTMS) for language mapping is largely unknown. Thus, to determine a minimum spatial resolution of rTMS for language mapping, we evaluated the mapping sessions derived from 19 healthy volunteers for cortical hotspots of no-response errors. Then, the distances between hotspots (stimulation points with a high error rate) and adjacent mapping points (stimulation points with low error rates) were evaluated.

**Results:**

Mean distance values of 13.8 ± 6.4 mm (from hotspots to ventral points, range 0.7–30.7 mm), 10.8 ± 4.8 mm (from hotspots to dorsal points, range 2.0–26.5 mm), 16.6 ± 4.8 mm (from hotspots to apical points, range 0.9–27.5 mm), and 13.8 ± 4.3 mm (from hotspots to caudal points, range 2.0–24.2 mm) were measured.

**Conclusions:**

According to the results, the minimum spatial resolution of rTMS should principally allow for the identification of a particular gyrus, and according to the literature, it is in good accordance with the spatial resolution of direct cortical stimulation (DCS). Since measurement was performed between hotspots and adjacent mapping points and not on a finer-grained basis, we only refer to a minimum spatial resolution. Furthermore, refinement of our results within the scope of a prospective study combining rTMS and DCS for resolution measurement during language mapping should be the next step.

## Background

Navigated transcranial magnetic stimulation (nTMS) nowadays plays a crucial role in presurgical planning, as it can be used to map cortical areas associated with motor or language function [1–5]. When applied with high frequency during an object-naming task (repetitive nTMS = rTMS), this technique is able to elicit a transient impairment of language or speech performance within the scope of cortical mapping [6–9].

Although language mapping based on rTMS is frequently used for preoperative neurosurgical diagnostics as well as neuroscientific trials, the spatial resolution of this method is largely unknown [1, 4–6, 10]. In this context, the spatial resolution of rTMS is regarded as the average of distances between language-positive and language-negative stimulation points. Since direct comparison to intraoperative stimulation by direct cortical stimulation (DCS), which is able to differentiate between essential, language-positive and language-negative areas, is not possible in healthy subjects, the present study was designed to assess the minimum spatial resolution of rTMS for language mapping. This was achieved by differentiating between rTMS hotspots (stimulation points with high naming error rates due to rTMS) and adjacent rTMS mapping points (stimulation points with low naming error rates), followed by discussion of results with regard to previous data derived from studies that used pre- or intraoperative mapping techniques. Regarding the stimulation approach, rTMS was carried out over predefined spots distributed across the left hemisphere, which follows the most common approach of rTMS-based language mapping to date.

## Methods

### Volunteers

All mapping sessions were originally performed in 19 healthy, right-handed (as indicated by the Edinburgh Handedness Inventory = EHI) subjects in 2013 to investigate different research questions of rTMS-based language mapping [11, 12]. The enrolled volunteers were German native speakers without general transcranial magnetic stimulation (TMS) exclusion criteria (e.g., metal implants).

### Magnetic resonance imaging

All imaging was performed on a magnetic resonance scanner (Achieva 3T, Philips Medical Systems, The Netherlands B.V.) by the use of an eight-channel phased-array head coil. Our scanning protocol consisted of a three-dimensional (3-D) gradient echo sequence (TR/TE 9/4 ms, 1 mm^3^ isovoxel covering the whole head, 6 min and 58 s acquisition time) without intravenous contrast administration. Subsequent to imaging, the 3-D magnetic resonance imaging (MRI) dataset of each subject was transferred to the rTMS system via DICOM standard.

### Transcranial magnetic stimulation

In all volunteers, language mapping by rTMS was performed using the Nexstim eXimia NBS, version 4.3, combined with a NexSpeech^®^ module and a biphasic figure-of-eight stimulation coil (Nexstim Oy, Helsinki, Finland).

#### Task

During baseline testing and rTMS-based language mapping, volunteers participated in an object-naming task, which has been frequently used in previous rTMS investigations [2, 6–8, 10–12]. The task consisted of a total amount of 100 colored photographs, similar to the objects of the Snodgrass and Vanderwart picture set (1980) [11–13]. The photographs portrayed familiar living as well as non-living objects (e.g., banana, chair, snake), and had to be named in German as quickly and precisely as possible.

For baseline testing, the volunteers had to name all objects, and any delayed or misnamed objects were discarded. Baseline testing was carried out twice, meaning that the second run was performed with the remaining object stack immediately after the first testing, and the total number of naming errors was documented in the end. Consequently, only objects that were correctly named twice were used during language mapping. Thus, baseline testing was performed to be able to remove objects the individual participants were not familiar with, resulting in an individualized stack of images. During language mapping, incorporation of objects that the participants are not acquainted with could cause incorrect attribution of naming errors to stimulation effects rather than to object unfamiliarity, leading to imprecise results.

#### Procedure

The examination started with the determination of the individual resting motor threshold (RMT) by motor mapping of the cortical representation of the hand area [2, 6, 7, 10]. The volunteers sat in a comfortable chair with armrests, and electrodes (Neuroline 720, Ambu, Ballerup, Denmark) were placed over the abductor pollicis brevis (APB) and abductor digiti minimi (ADM) muscle of the right hand to be able to detect motor evoked potentials (MEPs) during continuous electromyography (EMG) recording. Furthermore, the reference electrode was placed at the elbow. Then, a coarse round of single-pulse stimulations was conducted to localize the spot with the highest MEP amplitude, the motor hotspot, which is usually found within the area of the hand knob [14]. During pulse application, the induced electrical field was oriented perpendicular to the precentral gyrus, and the RMT was then determined at the motor hotspot. In this context, the RMT was defined as the lowest stimulation intensity that elicits MEPs over 50 µV in amplitude in at least 50% of stimulation trials in a relaxed muscle [15]. According to the stimulation protocol, the exact stimulation intensity for later language mapping was adjusted with respect to the RMT.

**Fig. 1:**
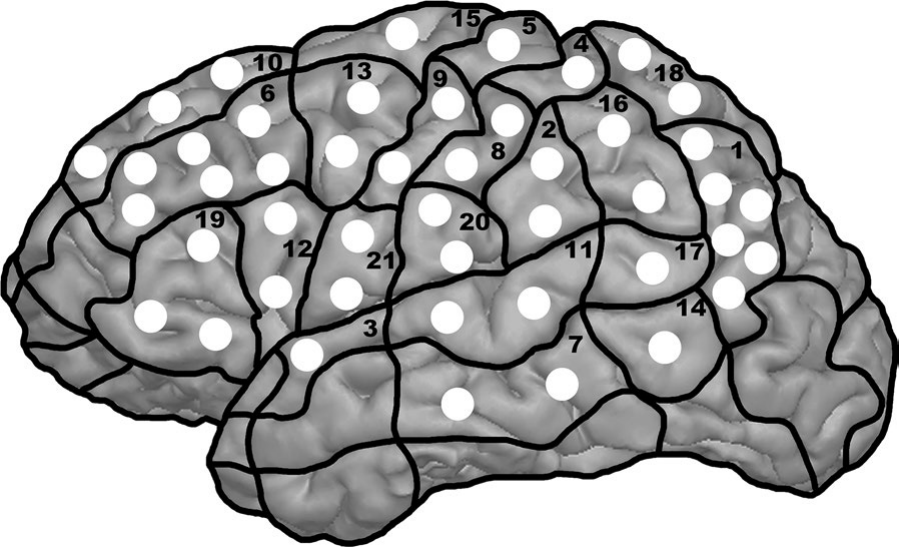
Cortical parcellation system (CPS). This figure visualizes the cortical stimulation targets (*white spots*, n = 46) within a template of the left hemisphere. In addition, the cortical surface is divided into subregions, and the numbers refer to the anatomical names of the stimulated subregions (see Table 1 for anatomical names and abbreviations of the CPS)

During stimulation, the subjects had to name their individualized sets of objects according to previous baseline testing. Thus, all objects that were correctly named twice according to baseline testing were displayed in randomized order while rTMS was applied in a time-locked fashion. Overall, 46 left-hemispheric cortical spots, which were manually tagged on the 3-D MRI scan of the volunteer prior to mapping, were stimulated three times each in a row without any particular order (Fig. 1), as reported previously [11, 12]. Thus, mapping was carried out over predefined spots.

Due to unacceptable stimulation-related discomfort, none of the 46 points were located within the following regions: orbital part of the inferior frontal gyrus (orIFG), polar superior temporal gyrus (polSTG), polar middle temporal gyrus (polMTG), anterior middle temporal gyrus (aMTG), polar superior frontal gyrus (polSFG), polar middle frontal gyrus (polMFG), and polar inferior frontal gyrus (polIFG). In addition, the inferior temporal gyrus (ITG) was also not mapped because of the increased coil-cortex distance. Parcellation of the anatomical structures was performed according to the cortical parcellation system (CPS, Table 1; Fig. 1) [2, 6, 16].

**Table 1:** Cortical parcellation system (CPS)

Number	Abbreviation	Anatomy
1	anG	Angular gyrus
2	aSMG	Anterior supramarginal gyrus
3	aSTG	Anterior superior temporal gyrus
4	dPoG	Dorsal postcentral gyrus
5	dPrG	Dorsal precentral gyrus
6	mMFG	Middle middle frontal gyrus
7	mMTG	Middle middle temporal gyrus
8	mPoG	Middle postcentral gyrus
9	mPrG	Middle precentral gyrus
10	mSFG	Middle superior frontal gyrus
11	mSTG	Middle superior temporal gyrus
12	opIFG	Opercular inferior frontal gyrus
13	pMFG	Posterior middle frontal gyrus
14	pMTG	Posterior middle temporal gyrus
15	pSFG	Posterior superior frontal gyrus
16	pSMG	Posterior supramarginal gyrus
17	pSTG	Posterior superior temporal gyrus
18	SPL	Superior parietal lobe
19	trIFG	Triangular inferior frontal gyrus
20	vPoG	Ventral postcentral gyrus
21	vPrG	Ventral precentral gyrus

Anatomical names and abbreviations of the CPS for all regions stimulated. The numbers refer to the individual subregions, which are visualized in Fig. 1

For transcranial stimulation, the magnetic coil was placed tangential to the subject’s skull, and the induced electrical field was oriented in strict anterior–posterior direction during language mapping [2, 6, 8, 17, 18]. Both the coil angulation (tangential to the subject’s skull) and electrical field orientation (anterior–posterior) were displayed by the help of the neuronavigation unit. Stimulation trials with incorrect coil angulation or orientation were repeated and not taken into account during analysis. All points of stimulation and the electrical field strength at the stimulation spots were automatically saved for later analysis. Moreover, a video of the naming performance during baseline testing as well as during stimulation was recorded for post hoc analysis.

#### Protocol

For each mapping session, the following stimulation parameters were chosen, as they (a) have shown to be efficient in terms of eliciting reproducible naming errors during object naming, and (b) have been proven to be well tolerable and safe for the individual subject [1, 2, 6–8, 10–12]:

- Stimulation intensity: 100% of the RMT
- Stimulation frequency: 7 Hz
- Number of pulses: 10
- Duration of each stimulation burst (7 Hz/10 pulses): 1430 ms
- Picture-to-trigger interval (=PTI, time between the presentation of an object on the screen and the beginning of the rTMS pulse): 0 ms
- Display time (=DT, duration of the screening of an object): 700 ms
- Inter-picture interval (=IPI, time between the screening of two objects): 3000 ms

### Data analysis

#### Error maps

All videos were analyzed for no-response errors strictly blinded to the stimulated cortical spots by the same person who had already conducted rTMS [6–8, 19]. A no-response error was defined as a complete lack of naming response within the duration of the stimulation. Other error types, like hesitations or performance errors, were not taken into account in the present investigation. Although other error types represent stimulation-induced disruption of important language subfunctions as well, we decided to not take these categories into account since no responses have proven to be among the most frequent error types whilst being easy to detect during video analysis [6, 20].

**Fig. 2:**
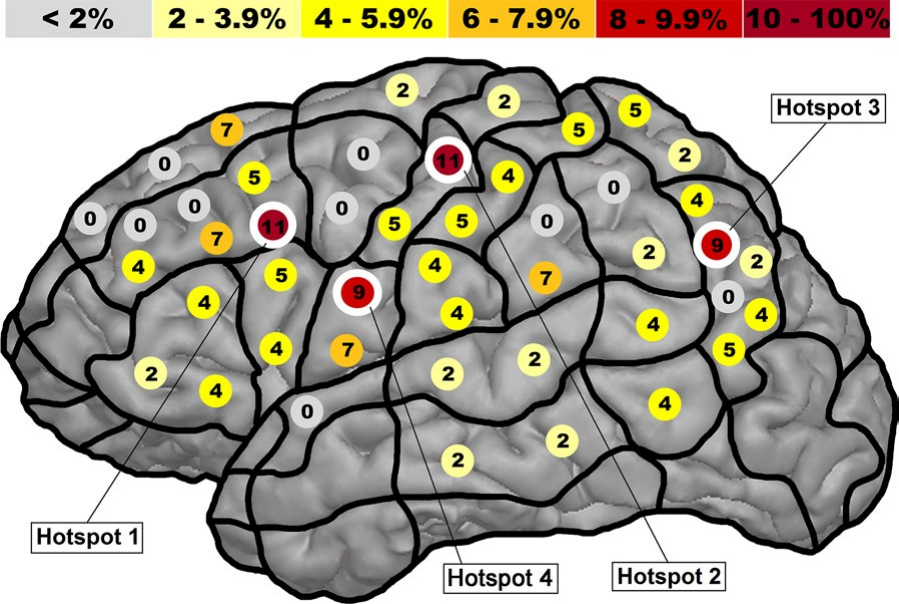
Distribution of no-response errors. This figure shows the no-response error rates (=number of induced no responses at a certain stimulation spot divided by the total number of stimulation bursts applied to this spot) as a percentage projected into the cortical parcellation system (CPS) including all stimulated spots (n = 46). Additionally, the four identified hotspots are marked

The total numbers of no-response errors as well as the numbers of stimulation bursts were pooled across all volunteers. Then, mapping results of the 46 stimulated cortical spots were projected into the CPS by putting pooled error rates (=number of induced no responses at one of the 46 cortical spots divided by the total number of stimulation bursts applied to this spot) on a brain template of the left hemisphere (Fig. 2).

#### Resolution measurement

Cortical spots that were prone to comparatively high no-response error rates (=hotspots) and were surrounded by four spots with lower error rates were identified from the raw data of mapping results and the error map (Fig. 2). Hence, hotspot definition was based on visual inspection of error distributions (Fig. 2) without additional statistical testing between single stimulation points. The four adjacent spots were separated into one ventral, one dorsal, one apical, and one caudal point with respect to the localization of the hotspot. If there were more than four points matching the inclusion criteria for being adequate adjacent spots, the closest one to the hotspot was chosen. Applying the described rule to Fig. 2, a number of four stimulated points fulfilled the hotspot criteria (hotspots 1, 2, 3, and 4). Hotspot 1, for example, shows a no-response error rate of 11%, and is surrounded by values of only 7% (ventral spot within the mMFG), 5% (apical spot within the mMFG), 0% (dorsal spot within the pMFG), and 5% (caudal spot within the opIFG). Each of these adjacent spots shows an error rate of less than 11% and can clearly be characterized as ventral, dorsal, apical, or caudal with respect to the hotspot.

**Fig. 3:**
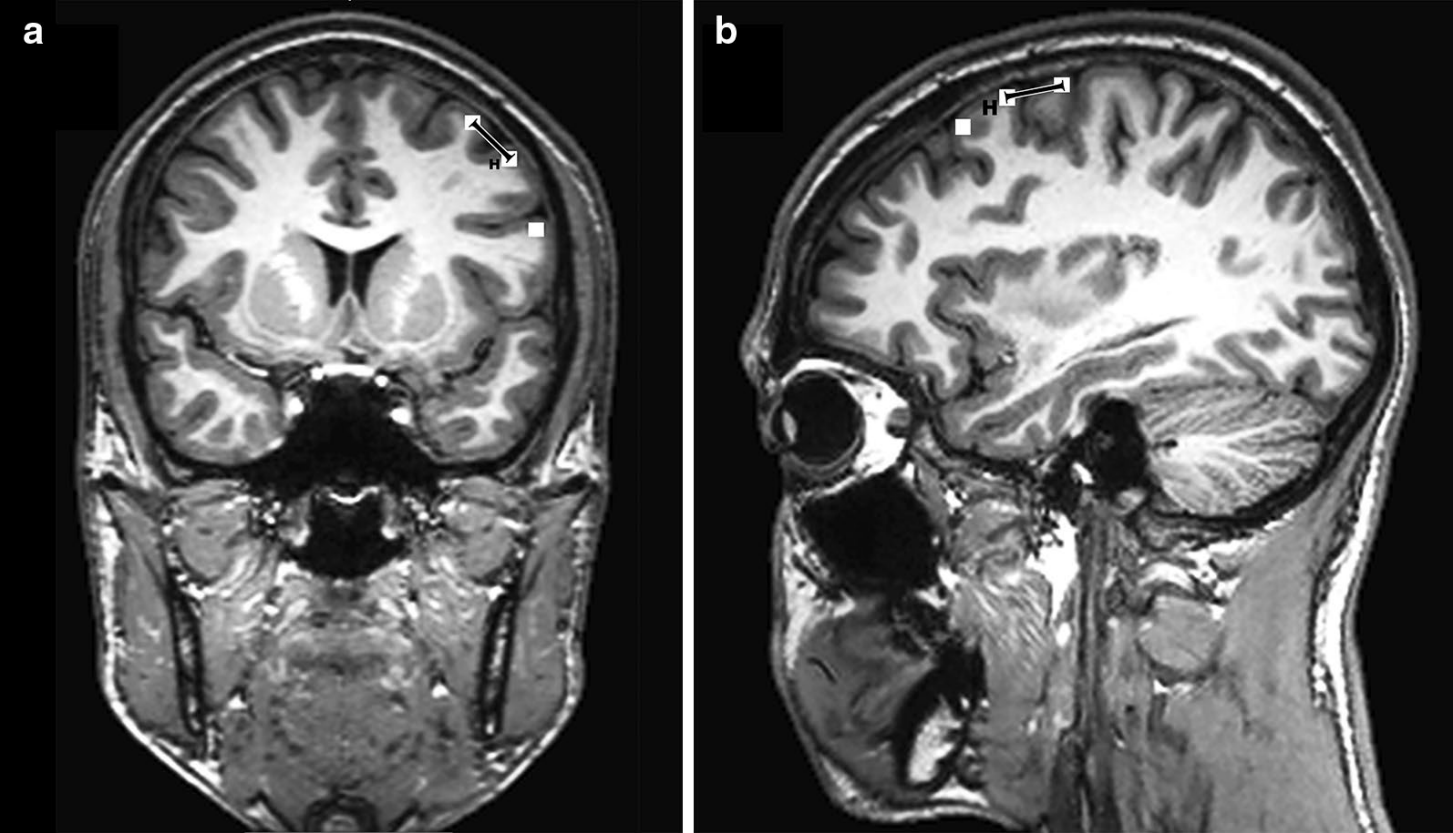
Spatial resolution measurement. Illustration of the distance measurement procedure on a subject’s individual coronal magnetic resonance imaging (MRI) slice for the apical and caudal points to the hotspot (**a**), and on a sagittal MRI slice for the distance between the hotspot and the ventral and dorsal spots (**b**). The *black lines* represent the distance between the hotspot (H) and the corresponding apical (**a**) or dorsal point (**b**) parallel to the cortical surface

After identification of the four suitable hotspots and their adjacent points on the template (Fig. 2), these spots were exported from each volunteer’s individual rTMS mapping session via DICOM standard to an external working station. Then, measurement of a minimum spatial resolution for rTMS-based language mapping was performed using OsiriX imaging software (OsiriX version 5.8.5, Pixmeo SARL, Bernex, Switzerland). Therefore, the two-dimensional (2-D) distances parallel to the cortical surface between each hotspot and its corresponding adjacent points were measured separately on the individual coronal MRI slices for the apical and caudal points to the hotspot (Fig. 3a), and on the sagittal MRI slices for the distance between the hotspot and the ventral as well as the dorsal spots (Fig. 3b). Since measurement was done between hotspots and adjacent mapping points belonging to the CPS and not on a finer-grained basis including cortical spots lying in-between, we refer to a minimum spatial resolution.

#### Statistics

Mean values ± standard deviation (SD), medians, minimum and maximum values of subject-related characteristics, mapping parameters, and distances between mapping spots were calculated by using GraphPad Prism (GraphPad Prism 6, La Jolla, CA, USA). Furthermore, a one-way analysis of variance (ANOVA) between the individual electrical field strengths of the enrolled subjects at the hotspots was performed, and a one-way ANOVA followed by Tukey’s multiple comparisons test, including calculation of 95% confidence intervals (CIs), was carried out to compare distance measurements between hotspots and adjacent mapping points. For all statistical calculations, a p value of <0.05 was considered statistically significant.

## Results

### Subject and mapping characteristics

Mappings were successfully performed in 19 healthy, right-handed volunteers, which were already analyzed for different purposes in previous investigations [11, 12]. Table 2 gives an overview of subject-related characteristics and mapping parameters. Regarding the comparison of electrical field strengths at the hotspots (Table 2), there was no statistically significant difference between the four hotspots (p = 0.3445).

**Table 2:** Subject and mapping characteristics

	Mean ± SD	Range
Age (years)	24.6 ± 1.7	21.8–29.4
EHI score	84.3 ± 13.2	57–100
Pain (VAS)		
Convexity	2.2 ± 1.6	0–6
Temporal	5.0 ± 2.0	2–10
Correct baseline objects	93.7 ± 3.3	87–98
RMT (% output)	33.5 ± 5.1	24–43
Electric field strength (V/m)		
Hotspot 1	62.0 ± 9.8	48–84
Hotspot 2	58.0 ± 11.5	40–83
Hotspot 3	63.0 ± 10.3	42–87
Hotspot 4	65.0 ± 14.1	42–90

Overview about subject-related characteristics including age, Edinburg Handedness Inventory (EHI) scores, and discomfort during stimulation according to the visual analogue scale (VAS). Moreover, the individual amount of correctly named objects during baseline testing, the resting motor threshold (RMT), and the electrical field strength that was applied to the four hotspots during stimulation are shown. In this context, the electrical field strength for each mapping point was automatically calculated and saved by the system

### Spatial resolution

**Fig. 4:**
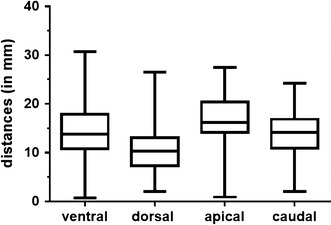
Measurement results. Boxplots with median, min-, and max-whiskers, and quartile-boxes for the hotspot to ventral, dorsal, apical, and caudal distance measurements (in mm)

Overall, mean distance values of 13.8 ± 6.4 mm (average of all hotspots to ventral points, range 0.7–30.7 mm), 10.8 ± 4.8 mm (average of all hotspots to dorsal points, range 2.0–26.5 mm), 16.6 ± 4.8 mm (average of all hotspots to apical points, range 0.9–27.5 mm), and 13.8 ± 4.3 mm (average of all hotspots to caudal points, range 2.0–24.2 mm) were measured. More detailed, Table 3 provides information about the mean ± SD, minimum, and maximum distance values of the measurements between each particular hotspot and the corresponding adjacent points. Furthermore, the results of measurements between all hotspots taken together and adjacent points are illustrated in Fig. 4.

**Table 3:** Measurement results

Hotspot	Adjacent spots	Mean	SD	MIN	MAX	p value
1	Ventral	11.9	2.2	8.6	16.8	<0.0001
	Dorsal	11.5	5.4	6.0	26.5	
	Apical	18.6	2.5	14.7	22.1	
	Caudal	14.4	2.8	9.0	17.8	
2	Ventral	18.0	3.9	11.6	25.8	<0.0001
	Dorsal	14.1	3.1	9.3	20.1	
	Apical	20.6	3.7	14.1	27.5	
	Caudal	17.7	2.9	13.8	24.2	
3	Ventral	10.7	8.3	0.7	30.7	<0.0001
	Dorsal	6.9	2.2	2.0	11.0	
	Apical	14.6	2.8	7.2	20.9	
	Caudal	12.3	3.0	6.5	18.2	
4	Ventral	14.5	6.8	1.9	27.5	0.1111
	Dorsal	10.7	4.9	3.8	18.4	
	Apical	12.6	5.1	0.9	21.0	
	Caudal	10.9	4.7	2.0	17.8	
All directions	1	14.1	4.4	6.0	26.5	–
	2	17.6	4.1	9.3	27.5	
	3	11.1	5.5	0.7	30.7	
	4	12.2	5.5	0.9	27.5	
All hotspots and directions		13.8	5.5	0.7	30.7	–

This table provides information about the spatial resolution measurements (in mm) by showing the distances from the four different hotspots to the four corresponding adjacent points (hotspot to ventral, dorsal, apical, and caudal spots), presented as mean ± standard deviation (SD), minimum (MIN), and maximum (MAX) values. In addition, one-way analysis of variance (ANOVA) was performed to compare distance measurements between hotspots and adjacent mapping points, leading to the p values presented in the table. In this context, a p value of <0.05 was considered statistically significant

According to ANOVA, statistically significant differences regarding the measurements between the hotspots and adjacent points were revealed with respect to hotspot 1, 2, and 3 (F = 16.5, F = 11.7, F = 8.8, p < 0.0001, Table 3), whereas no statistically significant difference was revealed concerning hotspot 4 (F = 2.1, p = 0.1111, Table 3). Regarding hotspot 1, there was a statistically significant difference in measurements for the hotspot to ventral versus apical points (CI −9.6 to −3.7), to dorsal versus apical points (CI −10.0 to −4.1), and to apical versus caudal points (CI 1.2 to 7.2), whereas statistically significant differences were revealed for the hotspot to ventral versus dorsal (CI 1.0 to 6.9), to dorsal versus apical (CI −9.5 to −3.6), and to dorsal versus caudal points (CI −6.6 to −0.7) for hotspot 2. Concerning hotspot 3, there was a statistically significant difference in measurements for the hotspot to dorsal versus apical points (CI −11.8 to −3.6) and to dorsal versus caudal points (CI −9.5 to −1.3).

## Discussion

### Current knowledge

Only a few publications investigated the resolution of TMS up to now [21–24]. The publications of Opitz et al. [21] and Thielscher et al. [22] calculated the electrical field induced by TMS in the human brain and came to the conclusion that the field strength is significantly enhanced when the currents run perpendicular to the stimulated gyrus. Moreover, this effect was demonstrated to be primarily restricted to the gyral lips and crowns, but it did not extend into the walls of the sulci, which means that the focality of the electrical field is increased [22]. The authors Bijsterbosch et al. [23] systematically investigated the impact of various TMS coil positions on electrical field shaping, finding that, at least for most coil positions, the induced field includes the target region of stimulation but is not distinctly restricted to it. According to their publication, the distribution of subarachnoid cerebral fluid and gyral geometry predominantly influence the modelling of the electrical field in the human brain [23].

Although the described publications represent extensive and valuable contributions to the current knowledge about the spatial resolution of TMS, they neither explicitly focused on cortical language mapping, nor did they examine the spatial resolution from a functional point of view, meaning that they did not particularly aim for a differentiation of functionally relevant from irrelevant cortical spots [21–23]. Under these premises, examination of the spatial resolution of rTMS-based language mapping seemed mandatory.

### Gyrus identification

The average width of a cortical gyrus in the adult human brain varies between 10 and 20 mm, although there can be distinct inter-individual variations. Therefore, when the central point of a figure-of-eight TMS coil is placed perpendicular to the subject’s skull and in the middle of a certain gyrus, the center of stimulation should primarily hit the stimulated gyrus, as our mean spatial resolution measurements for each of the four directions accounted for values clearly under 20 mm (Table 3; Fig. 4). As an important result, we can state that specific gyri can be targeted and identified by rTMS, at least when stimulation is applied to the gyrus center with the protocol used in the present study.

Furthermore, because the spatial resolution of rTMS does not exceed the average gyrus width and a particular gyrus can be targeted by this modality, the common practice of parcellating the cortical surface into subregions according to its gyral structure (e.g., as is done for the CPS) seems to be a reasonable approach for rTMS research.

### Electrical field strength

In general, a higher electrical field strength induced by rTMS should principally cause a lower spatial resolution and vice versa. The mean electrical field strengths at the four hotspots were comparable, and there was no statistically significant difference revealed. Thus, our approach including a comparison of hotspots being located within different remote cortical gyri seems reasonable because the applied electrical field does not significantly vary between these spots, and therefore, minimum measured distances seem comparable. However, statistical analysis of the four hotspots revealed a statistically significant difference of the mean distances between the hotspots and adjacent stimulation spots (Table 3), highlighting that the mean differences in distance measurements to the hotspot were different for the respective adjacent mapping points. This might show that spatial resolution of rTMS for language mapping—at least within a certain range—depends on the localization of cortical stimulation, although the applied electrical field strength does not significantly change during mapping. Yet, this effect might also be due to the chosen distribution of our cortical stimulation spots, and we can state that the minimally measured spatial resolution of rTMS was 11.1 ± 5.5 mm (Table 3).

### Comparison to other modalities

Overall, cortical language distribution has been under extensive investigation during the last decades. In this context, one frequently used technique for the identification of cortical sites related to language function is functional magnetic resonance imaging (fMRI). In its most common form, this approach compares the blood oxygenation level dependent (BOLD) signal during a language-related task performance to the signal obtained during resting-state measurement within the same subject [25, 26]. Then, the systematic comparison of both signal datasets allows drawing conclusions about the localization of individual language-related brain areas. Overall, fMRI is characterized by comparatively good spatial resolution, and a recent study on nTMS-based motor mapping has demonstrated that the spatial differences between nTMS- and DCS-positive cortical spots and fMRI- and DCS-positive points are both in the range of millimeters, although better results were observed for nTMS- versus DCS-positive spots (10.5 ± 5.7 vs. 15.0 ± 7.6 mm) [27].

Another neuroimaging modality for the investigation of cortical language representation is magnetoencephalography (MEG), which has been particularly used for determination of language lateralization over the last years [28, 29]. In this context, cortical activation in response to a language-related task performance goes along with a local rise in neuronal signaling, which is characterized by an increased flow of intracellular ions mediating associated magnetic fields that can be measured at the scalp surface in the form of event-related potentials [30]. However MEG has failed to show sufficient correlation with language maps generated by rTMS or DCS, at least partly due to its lower spatial resolution, which is typically limited to several centimeters [1, 31].

When it comes to DCS, which is regarded as the current gold standard for mapping human cortex function, language performance has been repeatedly tested during awake surgery [32–34]. Interestingly, Haglund et al. [33] showed that radical brain tumor resection without postoperative permanent language deficits can only be achieved reliably when the resection border is at least 10 mm away from the nearest language site determined by DCS. Thus, 10 mm seems to be the spatial resolution of DCS-based language mapping during awake surgery. As our findings show, the spatial resolution of rTMS-based language mapping is slightly above 10 mm (Table 3; Fig. 4), which is comparable to the results of DCS [33].

### Further considerations

Regarding the distribution of no responses across the hemisphere, the opIFG and trIFG, which should roughly overlap with the classical Broca’s area, were not characterized by a high rate of errors during rTMS. However, these regions showed error rates to a higher extent when it comes to other error categories, which were incorporated into analysis of the same dataset in other publications focusing on different purposes [11, 12]. Hence, considerable error rates were achieved during mapping of the opIFG and trIFG, but belonging to other types than no responses. As a possible explanation for the low rate of no responses in these regions when compared to previous investigations [6–8, 20], the stimulation frequency of 7 Hz (instead of 5 Hz) has to be considered, but the set of objects, which was different when compared to these previous publications, might have also played a role. However, the distinct cause for the specific pattern of no responses observed in the present study cannot be fully assessed within the scope of our approach.

Although the resolution measurements of the present study can be discussed in the light of previous literature on DCS-based language mapping, there is no opportunity to directly compare rTMS to DCS results on an intra-individual basis in healthy subjects due to the highly invasive character of DCS. As a consequence, the language-positive areas mapped in the present study cannot be verified by the gold standard method, and a final decision about the possible essential character of a particular language-positive site cannot be made due to the comparatively high sensitivity and low specificity of rTMS [10]. However, this limitation can at least partly be overcome by the results of recent studies that showed a much higher specificity and positive predictive value between language maps generated by both mapping techniques using the CPS, which again provides spatial resolution data on a gyri level [1, 2]. To draw more definite conclusions concerning resolution, a prospective study including preoperative rTMS and intraoperative DCS mapping among patients should be the next step.

In the recent years, the differentiation of naming errors into various predefined error categories has been demonstrated repeatedly within the scope of rTMS and DCS trials [4, 6, 8, 16, 20, 34]. Although other error categories except no responses are based on stimulation-induced disruption of important language subfunctions as well, we decided to not take these error types into account since no responses have proven to be among the most frequent error types [6, 20]. Incorporation of other error types could have led to different hotspots, but potentially on the basis of more unclear language disruption. Therefore, our current approach seems to be reasonable within the scope of one of the first studies regarding the exploration of rTMS-based language mapping resolution. However, we have to be aware of the fact that upcoming studies systematically analyzing other categories might further refine our minimum resolution measurements. Additionally, the spatial resolution of rTMS should also be evaluated for mapping of other cognitive functions since such approaches are currently emerging. In this context, non-invasive assessment of calculation functions by rTMS has been successfully performed recently, but spatial resolution for this purpose is vastly unknown [35].

Furthermore, it is already known that rTMS mapping results and electrical field shaping depend on various mapping parameters, like the stimulation frequency or the angulation and shape of the magnetic coil, for example [1, 11, 17, 36]. As a consequence, it cannot be the claim of the present study to define the definite spatial resolution of rTMS-based language mapping independently from setup factors. Instead, this study at least provides some practical orientation on the minimum spatial resolution for a commonly used mapping protocol, which has revealed to be reliable in terms of inducing transient errors during object naming while being well tolerable for the individual subject.

Another potential limitation could be the fact that distance measurement was only performed for predefined cortical points. In this context, the rTMS resolution measurement was limited by the distance between single stimulation points of the CPS, meaning that areas in-between the different stimulation spots were not systematically examined. Thus, the results presented in the current approach reflect a minimum resolution measurement. Consequently, it might be possible that the definite resolution of rTMS is finer-grained. Yet, all cortical points of stimulation were placed manually at the center of the gyri, meaning that finer-grained mapping might also have targeted sulci instead of cortical tissue, which is generally not regarded as adequate for the rTMS-based language mapping approach per se [6, 8]. We decided to follow the presented kind of approach because one major objective was to investigate the required cortical distance to distinguish between hotspots (stimulation points with a high error rate) and adjacent mapping points (stimulation points with low error rates) in a practical setup. Basically, this method is similar to the measurement presented in Haglund et al., which represents a key reference for the accuracy of DCS-based language mapping during awake surgery [33].

## Conclusions

The present study examined the spatial resolution of rTMS-based language mapping via distance measurement. In this context, rTMS is able to differentiate between hotspots (stimulation points with a high error rate) and adjacent mapping points (stimulation points with low error rates) at a minimum distance of 11.1 ± 5.5 mm as measured for a parietal hotspot. According to these distance measurement results, the spatial resolution of rTMS should principally allow for the identification of a particular gyrus as it is the case for DCS-based language mapping during awake surgery as the current gold standard.

## Abbreviations

2-D: two-dimensional
3-D: three-dimensional
aMTG: anterior middle temporal gyrus
ANOVA: analysis of variance
ADM: abductor digiti minimi
APB: abductor pollicis brevis
BOLD: blood oxygenation level dependent
CI: confidence interval
CPS: cortical parcellation system
DT: display time
DCS: direct cortical stimulation
EHI: Edinburgh Handedness Inventory
EMG: electromyography
fMRI: functional magnetic resonance imaging
IPI: inter-picture interval
ITG: inferior temporal gyrus
MEG: magnetoencephalography
MEP: motor evoked potential
MRI: magnetic resonance imaging
nTMS: navigated transcranial magnetic stimulation
orIFG: orbital part of the inferior frontal gyrus
polIFG: polar inferior frontal gyrus
polMFG: polar middle frontal gyrus
polMTG: polar middle temporal gyrus
polSFG: polar superior frontal gyrus
polSTG: polar superior temporal gyrus
PTI: picture-to-trigger interval
RMT: resting motor threshold
rTMS: repetitive navigated transcranial magnetic stimulation
SD: standard deviation
TMS: transcranial magnetic stimulation
VAS: visual analogue scale

## References

[1] Tarapore PE, Findlay AM, Honma SM, Mizuiri D, Houde JF, Berger MS, Nagarajan SS (2013). Language mapping with navigated repetitive TMS: proof of technique and validation. NeuroImage.

[2] Krieg SM, Tarapore PE, Picht T, Tanigawa N, Houde J, Sollmann N, Meyer B, Vajkoczy P, Berger MS, Ringel F (2014). Optimal timing of pulse onset for language mapping with navigated repetitive transcranial magnetic stimulation. NeuroImage.

[3] Picht T, Schulz J, Vajkoczy P (2013). The preoperative use of navigated transcranial magnetic stimulation facilitates early resection of suspected low-grade gliomas in the motor cortex. Acta Neurochir (Wien).

[4] Rosler J, Niraula B, Strack V, Zdunczyk A, Schilt S, Savolainen P, Lioumis P, Makela J, Vajkoczy P, Frey D (2014). Language mapping in healthy volunteers and brain tumor patients with a novel navigated TMS system: evidence of tumor-induced plasticity. Clin Neurophysiol.

[5] Picht T, Mularski S, Kuehn B, Vajkoczy P, Kombos T, Suess O (2009). Navigated transcranial magnetic stimulation for preoperative functional diagnostics in brain tumor surgery. Neurosurgery.

[6] Sollmann N, Tanigawa N, Ringel F, Zimmer C, Meyer B, Krieg SM (2014). Language and its right-hemispheric distribution in healthy brains: an investigation by repetitive transcranial magnetic stimulation. NeuroImage.

[7] Hernandez-Pavon JC, Makela N, Lehtinen H, Lioumis P, Makela JP (2014). Effects of navigated TMS on object and action naming. Front Hum Neurosci.

[8] Lioumis P, Zhdanov A, Makela N, Lehtinen H, Wilenius J, Neuvonen T, Hannula H, Deletis V, Picht T, Makela JP (2012). A novel approach for documenting naming errors induced by navigated transcranial magnetic stimulation. J Neurosci Methods.

[9] Pascual-Leone A, Gates JR, Dhuna A (1991). Induction of speech arrest and counting errors with rapid-rate transcranial magnetic stimulation. Neurology.

[10] Picht T, Krieg SM, Sollmann N, Rosler J, Niraula B, Neuvonen T, Savolainen P, Lioumis P, Makela JP, Deletis V (2013). A comparison of language mapping by preoperative navigated transcranial magnetic stimulation and direct cortical stimulation during awake surgery. Neurosurgery.

[11] Hauck T, Tanigawa N, Probst M, Wohlschlaeger A, Ille S, Sollmann N, Maurer S, Zimmer C, Ringel F, Meyer B (2015). Stimulation frequency determines the distribution of language positive cortical regions during navigated transcranial magnetic brain stimulation. BMC Neurosci.

[12] Hauck T, Tanigawa N, Probst M, Wohlschlaeger A, Ille S, Sollmann N, Maurer S, Zimmer C, Ringel F, Meyer B (2015). Task type affects location of language-positive cortical regions by repetitive navigated transcranial magnetic stimulation mapping. PLoS ONE.

[13] Snodgrass JG, Vanderwart M (1980). A standardized set of 260 pictures: norms for name agreement, image agreement, familiarity, and visual complexity. J Exp Psychol Hum Learn.

[14] Niskanen E, Julkunen P, Saisanen L, Vanninen R, Karjalainen P, Kononen M (2010). Group-level variations in motor representation areas of thenar and anterior tibial muscles: Navigated Transcranial Magnetic Stimulation Study. Hum Brain Mapp.

[15] Rossini PM, Barker AT, Berardelli A, Caramia MD, Caruso G, Cracco RQ, Dimitrijevic MR, Hallett M, Katayama Y, Lucking CH (1994). Non-invasive electrical and magnetic stimulation of the brain, spinal cord and roots: basic principles and procedures for routine clinical application. Report of an IFCN committee. Electroencephalogr Clin Neurophysiol.

[16] Corina DP, Loudermilk BC, Detwiler L, Martin RF, Brinkley JF, Ojemann G (2010). Analysis of naming errors during cortical stimulation mapping: implications for models of language representation. Brain Lang.

[17] Epstein CM, Lah JJ, Meador K, Weissman JD, Gaitan LE, Dihenia B (1996). Optimum stimulus parameters for lateralized suppression of speech with magnetic brain stimulation. Neurology.

[18] Ruohonen J, Karhu J (2010). Navigated transcranial magnetic stimulation. Neurophysiol Clin.

[19] Picht T, Schmidt S, Brandt S, Frey D, Hannula H, Neuvonen T, Karhu J, Vajkoczy P, Suess O (2011). Preoperative functional mapping for rolandic brain tumor surgery: comparison of navigated transcranial magnetic stimulation to direct cortical stimulation. Neurosurgery.

[20] Krieg SM, Sollmann N, Tanigawa N, Foerschler A, Meyer B, Ringel F (2016). Cortical distribution of speech and language errors investigated by visual object naming and navigated transcranial magnetic stimulation. Brain Struct Funct.

[21] Opitz A, Windhoff M, Heidemann RM, Turner R, Thielscher A (2011). How the brain tissue shapes the electric field induced by transcranial magnetic stimulation. NeuroImage.

[22] Thielscher A, Opitz A, Windhoff M (2011). Impact of the gyral geometry on the electric field induced by transcranial magnetic stimulation. NeuroImage.

[23] Bijsterbosch JD, Barker AT, Lee KH, Woodruff PW (2012). Where does transcranial magnetic stimulation (TMS) stimulate? Modelling of induced field maps for some common cortical and cerebellar targets. Med Biol Eng Comput.

[24] Walsh V, Cowey A (2000). Transcranial magnetic stimulation and cognitive neuroscience. Nat Rev Neurosci.

[25] Binder JR, Frost JA, Hammeke TA, Cox RW, Rao SM, Prieto T (1997). Human brain language areas identified by functional magnetic resonance imaging. J Neurosci.

[26] Binder JR (2011). Functional MRI is a valid noninvasive alternative to Wada testing. Epilepsy Behav.

[27] Forster MT, Hattingen E, Senft C, Gasser T, Seifert V, Szelenyi A (2011). Navigated transcranial magnetic stimulation and functional magnetic resonance imaging: advanced adjuncts in preoperative planning for central region tumors. Neurosurgery.

[28] Findlay AM, Ambrose JB, Cahn-Weiner DA, Houde JF, Honma S, Hinkley LB, Berger MS, Nagarajan SS, Kirsch HE (2012). Dynamics of hemispheric dominance for language assessed by magnetoencephalographic imaging. Ann Neurol.

[29] Hirata M, Goto T, Barnes G, Umekawa Y, Yanagisawa T, Kato A, Oshino S, Kishima H, Hashimoto N, Saitoh Y (2010). Language dominance and mapping based on neuromagnetic oscillatory changes: comparison with invasive procedures. J Neurosurg.

[30] Frye RE, Rezaie R, Papanicolaou AC (2009). Functional neuroimaging of language using magnetoencephalography. Phys Life Rev.

[31] Lev MH, Grant PE (2000). MEG versus BOLD MR imaging: functional imaging, the next generation?. AJNR Am J Neuroradiol.

[32] Tate MC, Herbet G, Moritz-Gasser S, Tate JE, Duffau H (2014). Probabilistic map of critical functional regions of the human cerebral cortex: Broca’s area revisited. Brain.

[33] Haglund MM, Berger MS, Shamseldin M, Lettich E, Ojemann GA (1994). Cortical localization of temporal lobe language sites in patients with gliomas. Neurosurgery.

[34] Sanai N, Mirzadeh Z, Berger MS (2008). Functional outcome after language mapping for glioma resection. N Engl J Med.

[35] Maurer S, Tanigawa N, Sollmann N, Hauck T, Ille S, Boeckh-Behrens T, Meyer B, Krieg SM (2016). Non-invasive mapping of calculation function by repetitive navigated transcranial magnetic stimulation. Brain Struct Funct.

[36] Bolognini N, Ro T (2010). Transcranial magnetic stimulation: disrupting neural activity to alter and assess brain function. J Neurosci.

